# Carbocisteine as a Modulator of Nrf2/HO-1 and NFκB Interplay in Rats: New Inspiration for the Revival of an Old Drug for Treating Ulcerative Colitis

**DOI:** 10.3389/fphar.2022.887233

**Published:** 2022-06-08

**Authors:** Amir Mohamed Abdelhamid, Mahmoud E. Youssef, Simona Cavalu, Gomaa Mostafa-Hedeab, Amal Youssef, Sara T. Elazab, Samar Ibrahim, Shady Allam, Rehab Mohamed Elgharabawy, Eman El-Ahwany, Noha A. Amin, Ahmed Shata, Osama A. Mohammed, Mahmoud Said Ibrahim Abdeldaiem, Ahmed Alhowail, Gaber El-Saber Batiha, Engy A. El-Mahmoudy, Maram Attia, Alaa Allam, Mona Y. Zaater, Mona M. Osman, Manar Nader, Aya Taha, Nada Abul Makarem, Sameh Saber

**Affiliations:** ^1^ Department of Pharmacology, Faculty of Pharmacy, Delta University for Science and Technology, Gamasa, Egypt; ^2^ Faculty of Medicine and Pharmacy, University of Oradea, Oradea, Romania; ^3^ Pharmacology Department and Health Research Unit, Medical College, Jouf University, Sakakah, Saudi Arabia; ^4^ Pharmacology Department, Faculty of Medicine, Beni-Suef University, Beni Suef, Egypt; ^5^ Medical Pharmacology Department, Faculty of Medicine, Cairo University, Giza, Egypt; ^6^ Department of Pharmacology, Faculty of Veterinary Medicine, Mansoura University, Mansoura, Egypt; ^7^ Department of Pharmacy Practice, Faculty of Pharmacy, Ahram Canadian University, Giza, Egypt; ^8^ Department of Pharmacology and Toxicology, Faculty of Pharmacy, Menoufia University, Menoufia, Egypt; ^9^ Pharmacology and Toxicology Department, Faculty of Pharmacy, Tanta University, Tanta, Egypt; ^10^ Department of Immunology, Theodor Bilharz Research Institute, Giza, Egypt; ^11^ Department of Haematology, Theodor Bilharz Research Institute, Giza, Egypt; ^12^ Department of Clinical Pharmacology, Faculty of Medicine, Mansoura University, Mansoura, Egypt; ^13^ Department of Clinical Pharmacy, Faculty of Pharmacy, Delta University for Science and Technology, Gamasa, Egypt; ^14^ Department of Clinical Pharmacology, Faculty of Medicine, Ain Shams University, Cairo, Egypt; ^15^ Department of Clinical Pharmacology, Faculty of Medicine, Bisha University, Bisha, Saudi Arabia; ^16^ Clinical Pharmacy Department, School of Pharmaceutical Sciences, Universiti Sains Malaysia, George Town, Malaysia; ^17^ Pharmacy Practice Department, Faculty of Pharmacy, Sinai University, Ismailia, Egypt; ^18^ Department of Pharmacology and Toxicology, College of Pharmacy, Qassim University, Buraidah, Saudi Arabia; ^19^ Department of Pharmacology and Therapeutics, Faculty of Veterinary Medicine, Damanhour University, Damanhour, Egypt; ^20^ Department of Biochemistry, Faculty of Pharmacy, Delta University for Science and Technology, Gamasa, Egypt

**Keywords:** carbocisteine, Nrf2/HO-1, NFκB, repositioning, colitis, acetic acid

## Abstract

Ulcerative colitis (UC), an inflammatory bowel disease, is a chronic condition of a multifaceted pathophysiology. The incidence of UC is increasing internationally. The current therapies for UC lack relative effectiveness and are associated with adverse effects. Therefore, novel therapeutic options should be developed. It has been well documented that modulating the Nrf2/NFκB is a promising therapeutic target in inflammation. Carbocisteine is a mucoregulatory medication and its efficacy in COPD was found to be more closely related to its antioxidant and anti-inflammatory properties. Carbocisteine has not yet been examined for the management of UC. Hence, our approach was to investigate the potential coloprotective role of carbocisteine in acetic acid-induced colitis in rats. Our results revealed that carbocisteine improved colon histology and macroscopic features and subdued the disease activity as well. Additionally, carbocisteine attenuated colon shortening and augmented colon antioxidant defense mechanisms via upregulating catalase and HO-1 enzymes. The myeloperoxidase activity was suppressed indicating inhibition of the neutrophil infiltration and activation. Consistent with these findings, carbocisteine boosted Nrf2 expression along with NFκB inactivation. Consequently, carbocisteine downregulated the proinflammatory cytokines IL-6 and TNF-α and upregulated the anti-inflammatory cytokine IL-10. Concomitant to these protective roles, carbocisteine displayed anti-apoptotic properties as revealed by the reduction in the Bax: BCL-2 ratio. In conclusion, carbocisteine inhibited oxidative stress, inflammatory response, and apoptosis in acetic acid-induced UC by modulating the Nrf2/HO-1 and NFκB interplay in rats. Therefore, the current study provides a potential basis for repurposing a safe and a commonly used mucoregulator for the treatment of UC.

## Introduction

Ulcerative colitis (UC) is a chronic condition that causes diffuse mucosal inflammation in the colon. In around 95% of instances, it includes the rectum and may progress proximally in a symmetrical, circular, and unbroken pattern to involve parts or all of the large intestine (Saber et al., 2021d; Mosli et al., 2021). Estimates of the prevalence of UC range from 7 to 246 per 100,000 individuals (Al-Horani et al., 2022). In Egypt, although there is limited data about the incidence of UC, some studies reported a marked increase in the frequency of UC which might pose a substantial social and economic burden on the government and health systems in the coming years. (Moussa et al., 2021). Colorectal cancer risk is observed to be increased in persons with long-term UC (Kanters and Liska, 2022). While not fully understood, the pathophysiology of UC is multifaceted and involves environmental variables, abnormal host immunological responses, and probable intestinal dysbiosis in genetically predisposed individuals. (Cardos et al., 2021).

5-aminosalicylic acid (5-ASA), corticosteroids, immunosuppressive medicines, antibiotics, and biological therapies are among the medical treatments available to treat UC. These drugs have the potential to heal active disease, prevent recurrence, and improve quality of life, but the relative efficacy of these competing therapies is unknown (Marian et al., 2010; Talley et al., 2011). For this reason, novel therapeutic techniques focusing on newly identified mechanisms should be developed to maximize efficacy and avoid side effects (Kutbi et al., 2021).

Carbocisteine (CRBST) is a mucoregulatory medication that is commonly used to clear accumulated airway secretions in patients suffering from acute and chronic respiratory disorders (Brown and Pharmacy, 1988). CRBST has the ability to operate as both a direct scavenger of ROS and an indirect antioxidant (Nogawa et al., 2009; Yoshida et al., 2009). In a large-scale multi-center clinical trial, it was discovered that CRBST is effective in lowering the rate of acute exacerbations and improving quality of life in patients with chronic obstructive pulmonary disease (COPD) (Zheng et al., 2008); its efficacy in COPD was found to be more closely related to its anti-oxidant and anti-inflammatory properties (Rahman and Macnee, 2012). Animal studies have proven the anti-inflammatory activity of CRBST in models of induced lung inflammation including numerous distinct cytokine profiles (Asti et al., 1995; Pena et al., 1999; Ishibashi et al., 2001; Wang et al., 2015). CRBST has the potential to be a promising therapeutic option for UC. As a result, the current investigation was carried out in order to assess this role in colitis in rats using the acetic acid animal model. Experimental colitis induced by acetic acid has been used extensively as a model for intestinal inflammatory disease. The acetic acid use provokes nonspecific inflammatory picture comparable with human UC with reproducible lesions. The comprehension of its accuracy as a model for human UC with regard to immunological alterations is attributed to the understanding of the cytokine expression pattern in the colon mucosa as well as the influence of endoanal infusion of acetic acid on the appearance and aggravation of consequent macroscopic lesions (Bertevello et al., 2005).

## Materials and Methods

### Drugs and Chemicals

CRBST was obtained from Amyria Pharmaceutical Industries, Alexandria, Egypt. Sigma-Aldrich (MO, United States) supplied the acetic acid (AA) and carboxymethyl cellulose (CMC).

### Animals

Adult male 6-week-old Sprague–Dawley rats weighing 230 ± 25 gm were bought from the Faculty of Pharmacy’s animal facility at the Delta University. The animals were housed in cages with six rats per cage and were given 2 weeks to acclimate before the study. During the experiment, standard environmental conditions (25°C, 45%–55% humidity, and light:dark cycles 12:12 h) were maintained. The experimental protocol was evaluated and approved by the Department of Pharmacology of the Faculty of Pharmacy at the Delta University for Science and Technology. All operations adhered to the institutional ethics committee’s rules for the use and handling of laboratory animals (Approval code number: 22921).

### Induction of Colitis Using Acetic Acid in Rats

Rats were fasted overnight with free access to water. They were subsequently anesthetized with intra-peritoneal administration of ketamine (50 mg/kg)/xylazine (10 mg/kg), and 2 ml of AA (3% vol/vol in 0.9% saline) were administered rectally into the distal colon using soft 6F polypropylene pediatric nutrition catheter lubricated with K–Y jelly (Reckitt, NJ, United States). Before removing the catheter, 2 ml of air were administered to disseminate AA throughout the colon. To avoid physical trauma, the catheter was then carefully withdrawn. Rats were placed in a supine Trendelenburg position for 30 s to prevent the solution from being expelled or escaping backward (El-Rous et al., 2021).

### Experimental Design

Following the acclimation period, rats were randomly assigned to one of five groups (Table 1): 1) Normal (n = 6), rats administered 2 ml of normal saline (NS) at the induction day (ID) intra-rectally (IR); 2) CRBST 500 (n = 6), rats administered CRBST (500 mg/kg/day, p. o. in two divided doses) for 2 days before the ID and continued for additional 5 days after ID; 3) UC, (n = 12), rats received 2 ml of 3% AA solution in NS IR at the ID; 4) UC/CRBST 250 (n = 12), rats administered CRBST (250 mg/kg/day, p. o. in two divided doses) for 2 days before induction of colitis and continued for additional 5 days after ID; 5) **UC/CRBST 500** (n = 12), rats administered CRBST (500 mg/kg/day, p. o. in two divided doses) for 2 days before induction of colitis and continued for additional 5 days after ID; CRBST were suspended in 0.5% aqueous solution of carboxymethyl cellulose (CMC) as vehicle. The CMC solution was administered to control groups at the same volume for 2 days before induction of colitis and continued for additional 5 days after ID. Animals were sacrificed 24 h after the last dose of CRBST and samples were collected.

**TABLE 1:** Experimental design and treatment protocol.

Exp. Groups	Days 1–2	Day 3 (induction day)	Days (4–8)	Day 9 (sacrifice day)
Normal (n = 6)	Vehicle	2 ml NS (I.R.)	Vehicle	——
CRBST 500 (n = 6)	CRBST (500 mg/kg) p.o. in two divided doses	CRBST (500 mg/kg) p.o. in two divided doses +2 ml normal saline (I.R.)	CRBST (500 mg/kg) p.o. in two divided doses	——
UC (n = 12)	Vehicle	2 ml of 3% AA solution in NS (I.R.)	Vehicle	——
UC/CRBST 250 (n = 12)	CRBST (250 mg/kg) p.o in two divided doses	CRBST (250 mg/kg) p.o. in two divided doses +2 ml of 3% AA solution in NS (I.R.)	CRBST (250 mg/kg) p.o. in two divided doses	——
UC/CRBST 500 (n = 12)	CRBST (500 mg/kg) p.o in two divided doses	CRBST (500 mg/kg) p.o. in two divided doses +2 ml of 3% AA solution in NS (I.R.)	CRBST (500 mg/kg) p.o in two divided doses	——

AA, acetic acid; CRBST, Carbocisteine; NS, Normal saline; I.R., Intra-rectal; p.o., per oral route; Vehicle, 0.5% CMC; UC, Ulcerative colitis

### Rational of Dosage Regimen

The dosage regimen of CRBST was adopted from a previous study in Wistar rats in which CRBST (125 and 250 mg/kg ×2/day) was administered for 25 days after 20 days of SO_2_ gas exposure (Ishibashi et al., 2004).

### Assessment of the Disease Activity Index (DAI)

A blinded investigator quantified the disease activity index (DAI) using a previously known approach to assess the extent of the produced UC (Palla et al., 2016). Simply, the DAI was quantified as a collective score as described in Table 2.

**TABLE 2:** Disease activity index (DAI) criteria.

Parameter	Evaluation Criteria	Score
Percentage body weight loss	None	0
	1–5%	1
	6–10%	2
	11–20%	3
	>20%	4
Diarrhea	Normal	0
	Loose stools	1–2
	Watery diarrhea	3–4
Bloody stool	Normal	0
	Slight bleeding	1–2
	Gross bleeding	3–4

### Tissue Collection, Assessment of Colon Weight/Length Ratio and Assessment of the Macroscopic Damage Index (MDI)

Colons were excised from anus to caecum, rinsed with phosphate-buffered saline (PBS), and dehumidified with filter paper before their weight and length were recorded for each animal. Then, the relative colon weight/length was determined. Blinded macroscopic damage index (MDI) was calculated for each animal as a collective score based on specified parameters as shown in Table 3 (Youssef et al., 2021a). Finally, distal colons were dissected into parts: One part was immediately preserved in RNAlater (Qiagen, Germany), another part was homogenized (10% w/v) and the supernatant was collected and stored for further analysis, and the last part was prepared as paraffin blocks using standard histological techniques for histopathological examinations (Slaoui and Fiette, 2011).

**TABLE 3:** Macroscopic damage index (MDI) criteria.

Macroscopic Features	Score
No macroscopic changes	0
Mucosal erythema only	1
Mild mucosal edema, slight bleeding or small erosions	2
Moderate oedema, slight bleeding ulcers or erosions	3
Severe ulceration, oedema and tissue necrosis	4

### Histopathological Examination of Colonic Tissue

Tissues from paraffin blocks were serially sliced into 4-μm slices with a microtome, mounted on glass slides, and stained with hematoxylin and eosin (H&E) (Youssef et al., 2021b). A blinded semiquantitative histopathological evaluation was conducted using previously recognized criteria (Youssef et al., 2021a) (Table 4).

**TABLE 4:** Histological scoring system (microscopic evaluation of inflammation).

Microscopic Features	Score
No signs of inflammation	0
Very low level of inflammation	1
Low level of leukocyte infiltration	2
High level of leukocyte infiltration, high vascular density, thickening of the colon wall	3
Transmural infiltration, loss of goblet cells, high vascular density, thickening of the colon wall	4

### Biochemical Assessment of Catalase, Total Antioxidant Capacity (TAC) and Myeloperoxidase (MPO) Activity

Catalase (Cat. No. CA2517), and total antioxidant capacity (TAC, Cat. No. TA 2513) were assessed spectrophotometrically in colonic tissue homogenate using commercial kits supplied by Bio-diagnostic (Giza, Egypt). Myeloperoxidase (MPO) activity (Cat. No. MAK068-1 KT) was assessed using a kit supplied by Sigma-Aldrich (MO, United States), where MPO catalyzes the formation of hypochlorous acid, which reacts with taurine to form taurine chloroamine. A colorless product, DTNB, is formed when taurine chloroamine reacts with the chromophore.

### Assessment of TNF-α, IL-6, IL-10 and TLR4

According to the manufacturer’s instructions and using ELISA kits, interleukin-6 (IL-6, Cat. No. R6000B, R&D System, MN, United States), tumor necrosis factor-alpha (TNF-α, Cat. No. LS-F24977, LifeSpanBioSciences Inc., WA, United States), interleukin-10 (IL-10, Cat. No. MBS764911, MyBioSource (CA, United States) and toll-like receptor 4 (TLR4, Cat. No. SEA753Ra, Cloud-Clone Corp USCN Life Science Inc., Wuhan, China). were quantified. Colon tissues were homogenized in ice-cold buffer solution (PBS, pH 7.4) containing ethylenediaminetetraacetic acid (EDTA) and Tris-HCl with a glass homogenizer. The resulting suspension was sonicated using a Branson Sonifier (250, Danbury, CT, United States). The homogenates were centrifuged for 5 min at 5000×g., and supernatants were stored at −80 °C for subsequent analysis.

### Assessment of HO-1, BCL-2, Bax, NFκB, IκB-α, Nrf2, NFκB Nuclear DNA Binding Activity and Active Caspase-3

The concentration of heme oxygenase-1 (HO-1, Cat. No. ADI-EKS-810A) was determined using ELISA assay kits supplied from EnzoLifeSciences Inc. (Loerrach, Germany). B-cell lymphoma 2 (BCL-2, Cat. No. CSB-E08854r) concentrations were determined using CUSABIO ELISA test kits (Wuhan, China). The concentration of BCL2-associated X protein (Bax, Cat. No. E4513) was determined using an ELISA kit bought from Biovision Inc. (CA, United States). ELISA also was used to determine the phosphorylated NFκB p65/p65 ratio using commercial kits obtained from Abcam (Cat. No. ab176663) in which colons were homogenized in the provided extraction buffer and centrifuged at 18000 xg at 4°C; then the pellets were discarded and supernatants were stored at −80°C. IκB-α concentration was determined using ELISA commercial kits obtained from Fine Biotech (Wuhan, China, Cat. No. ER1084). Nuclear factor erythroid 2-related factor 2 (Nrf2, Cat. No. MBS012148) concentrations were determined using MyBioSource ELISA kits. All protocols were performed in accordance with the manufacturer’s guidelines. An assay kit obtained from abcam was used for the assessment of nuclear translocation of p65 subunit in nuclear extracts in which a specific double stranded DNA sequence containing the NF-κB p65 consensus binding site (5′–GGGACTTTCC–3′) binds to the active NF-κB p65 which is detected by a primary antibody that recognizes an epitope of NF-κB p65 accessible only when the protein is activated and bound to its target DNA (Abdelhamid et al., 2021b). The NF-κB p65 activity was determined in duplicate. The intrinsic cell death pathway is governed by the BCL-2 family of proteins (such as Bax AND BCL-2), which regulate commitment to cell death through the mitochondria. The key step in the intrinsic cell death pathway is the permeabilization of the mitochondrial outer membrane, after which cells are committed to cell death. Following permeabilization, the release of proteins from the mitochondrial intermembrane space promotes caspase activation and apoptosis. Released cytochrome C induces the activation of caspase-9. Caspase-9 then activates caspases-3, leading to apoptosis. Active caspases-3 was determined by a kit that was supplied by MyBioSource Inc. (San Diego, CA, United States) in which a competitive enzyme immunoassay technique utilizing a polyclonal anti-active caspase-3 antibody and an active caspase-3-HRP conjugate was used. The intensity of the color is inversely proportional to the active caspase-3 concentration since active caspase-3 from samples and active caspase-3-HRP conjugate compete for the anti-active caspase-3 antibody binding site. Since the number of sites is limited, as more sites are occupied by active caspase-3 from the sample, fewer sites are left to bind active caspase-3-HRP conjugate (Saber et al., 2021b). The active caspase-3 was determined in duplicate.

### Quantitative Real-Time PCR for the Expression of *H O -1*, *NFκB p65*, and *IκB-α* in Colon Tissue

The RNeasy Mini kit (QIAGEN, Germany) was used to isolate total RNA from colon tissue, and the QuantiTect Reverse Transcription Kit (QIAGEN, Germany) was used to reverse-transcribe the RNA into cDNA. The target gene was then amplified using primer sequences for the target genes listed in Table 5, SYBR Green Master Mix (Yeasen Biotech, China), and a thermocycler Rotor-Gene Q (Hilden, Germany), with each sample repeated three times. The relative expression of target gene mRNA compared to GAPDH was calculated using the 2^–ΔΔCt^ method.

**TABLE 5:** Primer sequences for qRT-PCR.

Gene	GenBank Accession	F	R	Amplicon Size (Bp)
NF-κB p65	NM_199267.2	5′- TTC​CCT​GAA​GTG​GAG​CTA​GGA -3′	5′- CAT​GTC​GAG​GAA​GAC​ACT​GGA -3′	185
IκBα	NM_001105720.2	5′- AAG​GAC​GAG​GAT​TAC​GAG​CAG -3′	5′- CCC​TTC​ACC​TGA​CCA​ATC​ACT -3′	185
HO-1	NM_012580.2	5′- GAGCCAGCCTGAACTAGC -3′	5′- GATGTGCACCTCCTTGGT -3′	107
GAPDH	NM_017008.4	5′-TCA​AGA​AGG​TGG​TGA​AGC​AG-3′	5′-AGG​TGG​AAG​AAT​GGG​AGT​TG-3′	111

### Statistical Analysis

The GraphPad Prism program (ver. 9.3.1, GraphPad Software Inc., CA, United States) was used for the statistical analysis. Parametric results are displayed as the mean ± standard deviation (SD), and were compared using one-way analysis of variance (ANOVA) followed by Tukey’s Kramer multiple comparison test. DAI, MDI, and histopathology scores are displayed as medians ± interquartile ranges and were compared using Kruskal–Wallis followed by Dunn’s multiple comparison test. The threshold for significance was set at *p <* 0.05.

## Results

### Effect of CRBST on Colon Weight/Length Ratio, Disease Activity Index (DAI) and Macroscopic Damage Index (MDI) in Rats With AA-Induced Colitis

**FIGURE 1:**
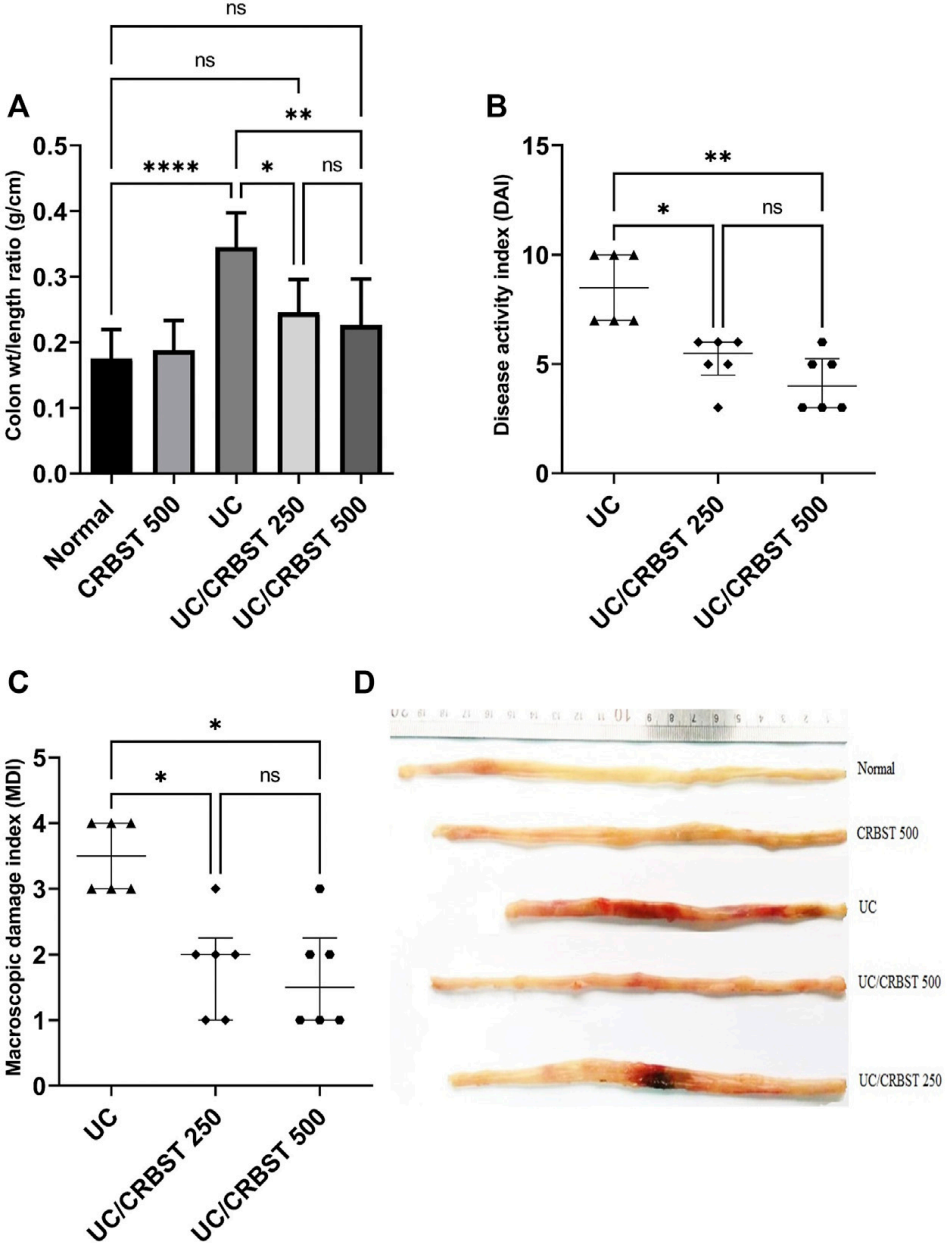
Effect of CRBST 250 and 500 mg/kg on colon weight/length ratio **(A)**; DAI **(B)**; MDI **(C)**; and the colon pictures **(D)** in rats with AA-induced UC. Results in figure **(A)** are shown as the mean ± SD and in figure **(B,C)** are shown as the median ± interquartile range (n = 6). ∗*p* < 0.05, ∗∗*p* < 0.01, ∗∗∗∗*p <* 0.0001. Normal, normal control rats administered the vehicle; CRBST 500, normal rats administered carbocisteine (500 mg/kg); UC, AA-induced UC rats administered the vehicle; UC/CRBST 250, AA-induced UC rats treated with carbocisteine (250 mg/kg); UC/CRBST 500, AA-induced UC rats treated with carbocisteine (500 mg/kg).

The physical examination of colon weight/length ratio showed that acetic acid (AA) significantly increased colon weight/length ratio in UC group when compared to normal group (Figure 1A). On the other hand, upon treatment with CRBST 250 or 500 mg/kg colon weight/length ratios were significantly decreased when compared to UC group. Also, the DAI (Figure 1B) and MDI (Figure 1C) were significantly decreased in CRBST-treated groups. The higher dose of CRBST, 500 mg/kg, was not superior over the 250 mg/kg dose. Figure 1D visualizes colon changes in terms of macroscopic features and colon length.

### Effect of CRBST on Histopathological Characteristics and Histopathological Score in Rats With AA-Induced Colitis

**FIGURE 2:**
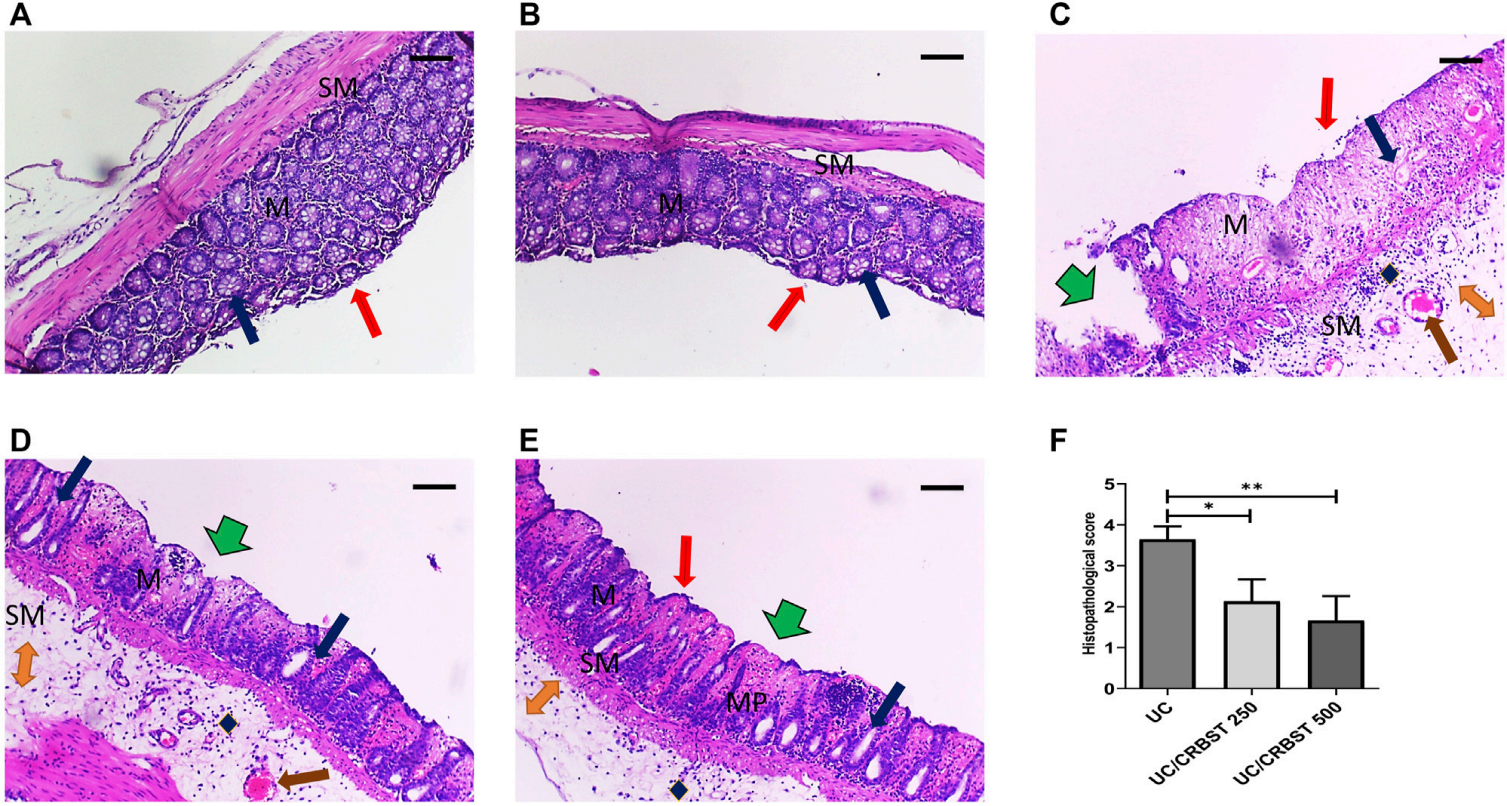
Effect of CRBST 250 and 500 mg/kg on histopathological characteristics and histopathological score in rats with AA-induced UC. Representative histological appearance of colon tissue specimens stained with H&E from Normal **(A)** and CRBST 500 **(B)** control groups showing normal epithelium (red arrow), normal colonic mucosa (M) and submucosa (SM), crypts and glands (blue arrow); Colonic sections from UC group **(C)** showing deepithelialization (red arrow), erosions (green arrow), disrupted mucosa (M) and submucosa (SM), inflammatory cell infiltration (blue diamond), edema (orange double arrow), congestion (brown arrow) and complete necrosis of the crypts (blue arrow); Colonic sections from UC/CRBST 250 **(D)** and UC/CRBST 500 **(E)** showing a moderate restoration of architecture, with decreased superficial ulceration of the intestinal mucosa (green arrow) and is associated with a marked decrease of interstitial inflammatory cell infiltration in (blue diamond), lower degree of glands damage (blue arrow), lower degree of edema (orange double arrow), normal muscularis propria (MP), congestion still apparent (brown arrow). Results in **(F)** are shown as the median ± interquartile range (n = 6). ***p < 0.001. Normal, normal control rats administered the vehicle; CRBST 500, normal rats administered carbocisteine (500 mg/kg); UC, AA-induced UC rats administered the vehicle; UC/CRBST 250, AA-induced UC rats treated with carbocisteine (250 mg/kg); UC/CRBST 500, AA-induced UC rats treated with carbocisteine (500 mg/kg). H&E stain, X 100, bar = 200 µm.

Photomicrographs of colon tissue stained with H&E from the Normal **(** Figure 2A) and CRBST 500 (Figure 2B) control groups showed normal colonic mucosa, crypts and glands. In contrast, the colon tissue from the UC group (Figure 2C) shows inflammatory-cell infiltration, edema and complete necrosis of the crypts. Upon treatment with CRBST (250 or 500 mg/kg) (Figures 2D,E, respectively), the AA-administered rats showed a moderate restoration of architecture, with decreased superficial ulceration of the intestinal mucosa and is associated with a marked decrease of interstitial inflammatory-cell infiltration. Moreover, treatment with CRBST 500 mg/kg resulted in a marked reduction in the histopathological score when compared with UC group (Figure 2F).

### Effect of CRBST on Catalase, TAC, MPO Activity and HO-1 in Rats With AA-Induced Colitis

**FIGURE 3:**
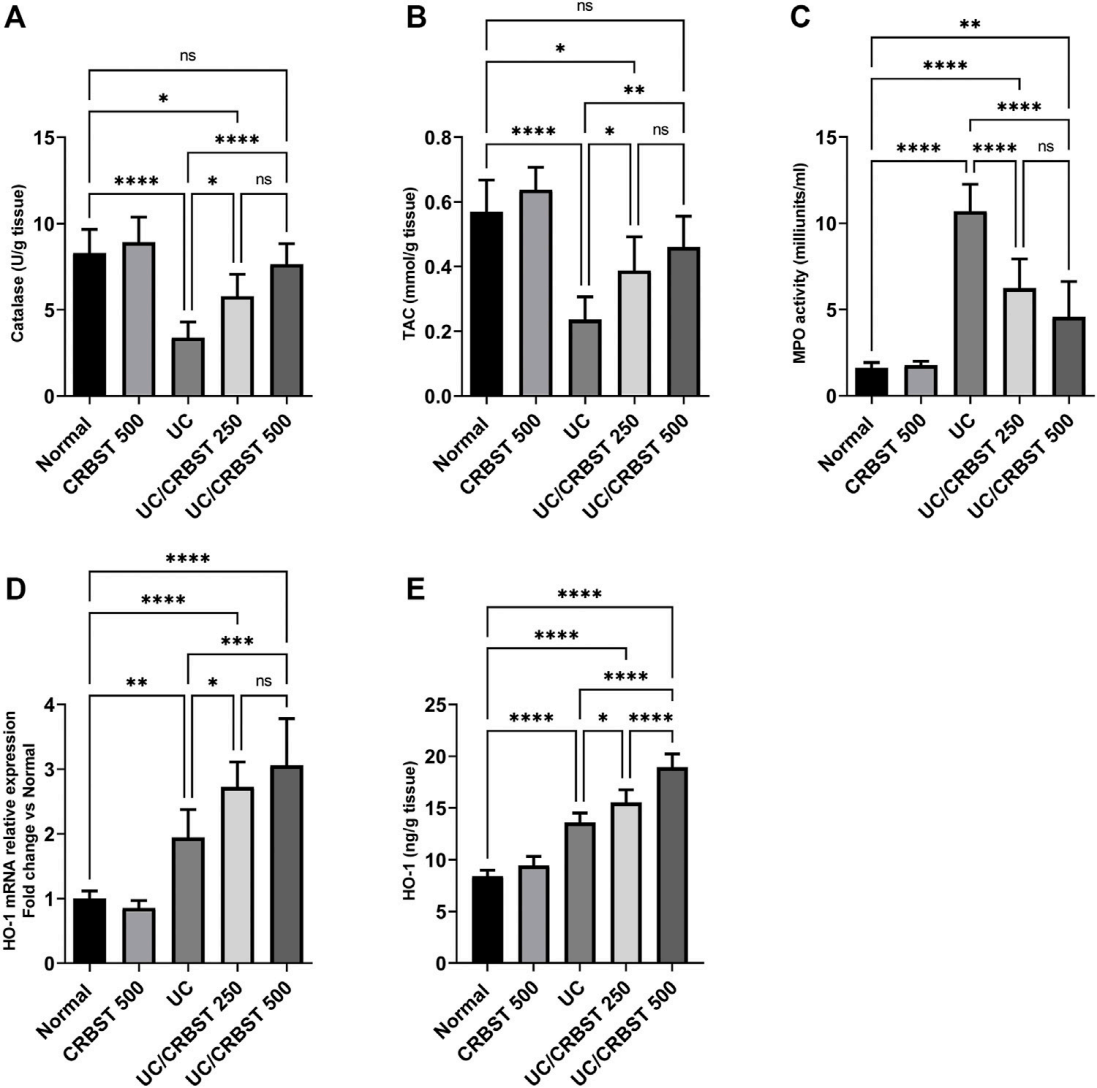
Effect of CRBST 250 and 500 mg/kg on catalase **(A)**; TAC **(B)**; MPO activity **(C)**; HO-1 mRNA **(D)**; and HO-1 **(E)** in rats with AA-induced UC. Results are shown as the mean ± SD (n = 6). *p < 0.05, **p < 0.01, ***p < 0.001, ****p < 0.0001. Normal, normal control rats administered the vehicle; CRBST 500, normal rats administered carbocisteine (500 mg/kg); UC, AA-induced UC rats administered the vehicle; UC/CRBST 250, AA-induced UC rats treated with carbocisteine (250 mg/kg); UC/CRBST 500, AA-induced UC rats treated with carbocisteine (500 mg/kg).

When compared to the normal group, AA-administration significantly decreased the colon tissue level of catalase (Figure 3A) and TAC (Figure 3B) with concomitant increase in MPO activity (Figure 3C) in UC group, indicating an increase in the oxidative stress in colon tissue; In addition, it significantly increased HO-1 expression in both gene expression (Figure 3D) and protein level (Figure 3E). Treatment with CRBST 250 or 500 mg/kg, on the other hand, significantly increased the tissue levels of catalase, TAC and HO-1, as well as, decreased the colonic MPO activity when compared to the UC group.

### Effect of CRBST on IL-6, TNF-α, IL-10 and TLR4 in Rats With AA-Induced Colitis

**FIGURE 4:**
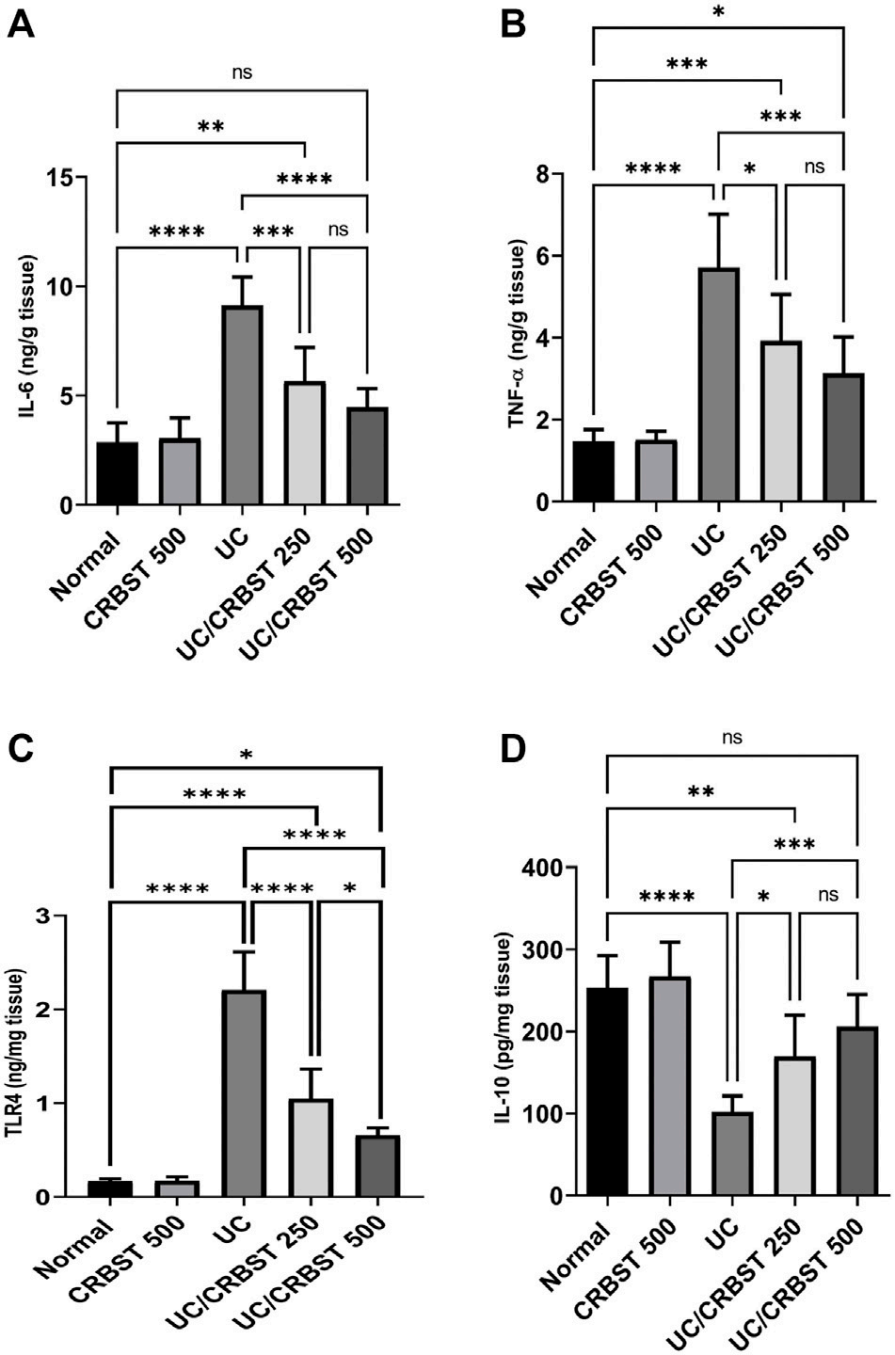
Effect of CRBST 250 and 500 mg/kg on IL-6 **(A)**; TNF-α **(B)**; TLR4 **(C)** and IL-10 **(D)** in rats with AA-induced UC. Results are shown as the mean ± SD (n = 6). *p < 0.05, **p < 0.01, ***p < 0.001, ****p < 0.0001. Normal, normal control rats administered the vehicle; CRBST 500, normal rats administered carbocisteine (500 mg/kg); UC, AA-induced UC rats administered the vehicle; UC/CRBST 250, AA-induced UC rats treated with carbocisteine (250 mg/kg); UC/CRBST 500, AA-induced UC rats treated with carbocisteine (500 mg/kg).

The administration of AA in UC group markedly increased the colonic content of IL-6 (Figure 4A), TNF-α (Figure 4B), and TLR4 (Figure 4C), and conversely decreased its IL-10 content (Figure 4D) compared to the normal group. In comparison with the UC group, treatment with CRBST 250 or 500 mg/kg to the AA-administered rats significantly decreased the tissue content of IL-6, TNF-α and TLR4 and significantly increased the IL-10 content. Using the 500 mg/kg dose of CRBST was not superior to the 250 mg/kg dose in improving IL-6, TNF-α and IL-10 content. However, using the 500 mg/kg dose of CRBST was superior to the 250 mg/kg dose in downregulating the TLR4 (*p* < 0.05).

### Effect of CRBST on Bax, BCL-2 and Active Caspase-3 in Rats With AA-Induced Colitis

**FIGURE 5:**
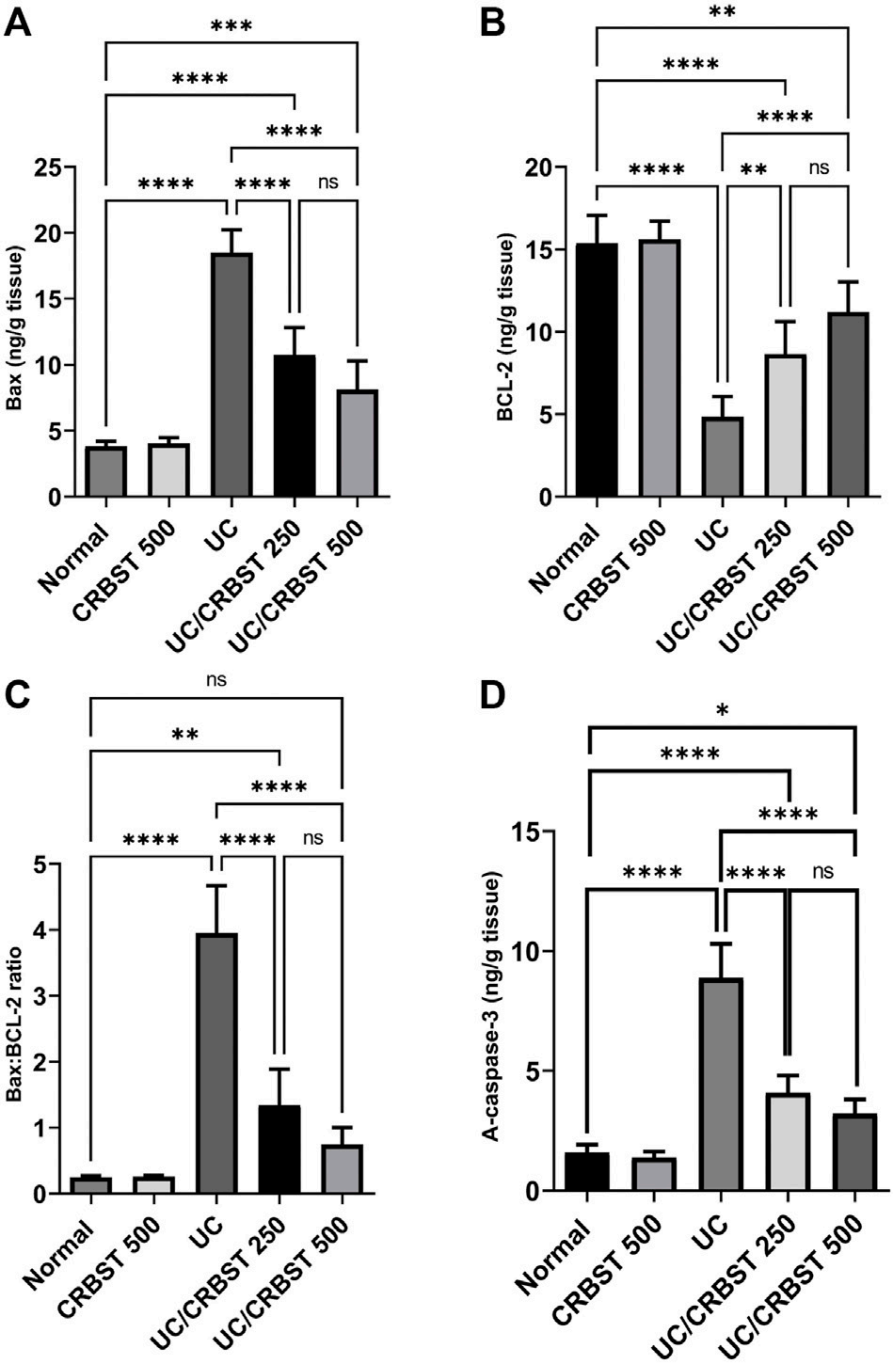
Effect of CRBST 250 and 500 mg/kg on Bax **(A)**; BCL-2 **(B)**; Bax: BCL-2 ratio **(C)** and A-caspase-3 **(D)** in rats with AA-induced UC. Results are shown as the mean ± SD (n = 6). **p < 0.01, ***p < 0.001, ****p < 0.0001. Normal, normal control rats administered the vehicle; CRBST 500, normal rats administered carbocisteine (500 mg/kg); UC, AA-induced UC rats administered the vehicle; UC/CRBST 250, AA-induced UC rats treated with carbocisteine (250 mg/kg); UC/CRBST 500, AA-induced UC rats treated with carbocisteine (500 mg/kg).

Compared to the healthy rats in the normal group, rats in the UC group showed a marked increase in Bax (Figure 5A) and a marked decrease in BCL-2 (Figure 5B) content which resulted in a significant increase in Bax: BCL-2 ratio (Figure 5C), indicating an increase in the apoptotic activity. In contrast, the Bax levels decreased, the BCL-2 levels increased and Bax: BCL-2 ratio decreased significantly upon treatment with CRBST 250 or 500 mg/kg. There was no significant difference between both doses of CRBST. A-caspase-3 levels (Figure 5D) were consistent with the Bax: BCL-2 ratio. In this regard both doses revealed lower levels in the UC rats compared to the untreated UC rats. Using the 500 mg/kg dose of CRBST was not superior to the 250 mg/kg dose in its antiapoptotic potential.

### Effect of CRBST on NFκB p-65, IκBα, Nrf2 and NFκB Nuclear DNA Binding Activity in Rats With AA-Induced Colitis

**FIGURE 6:**
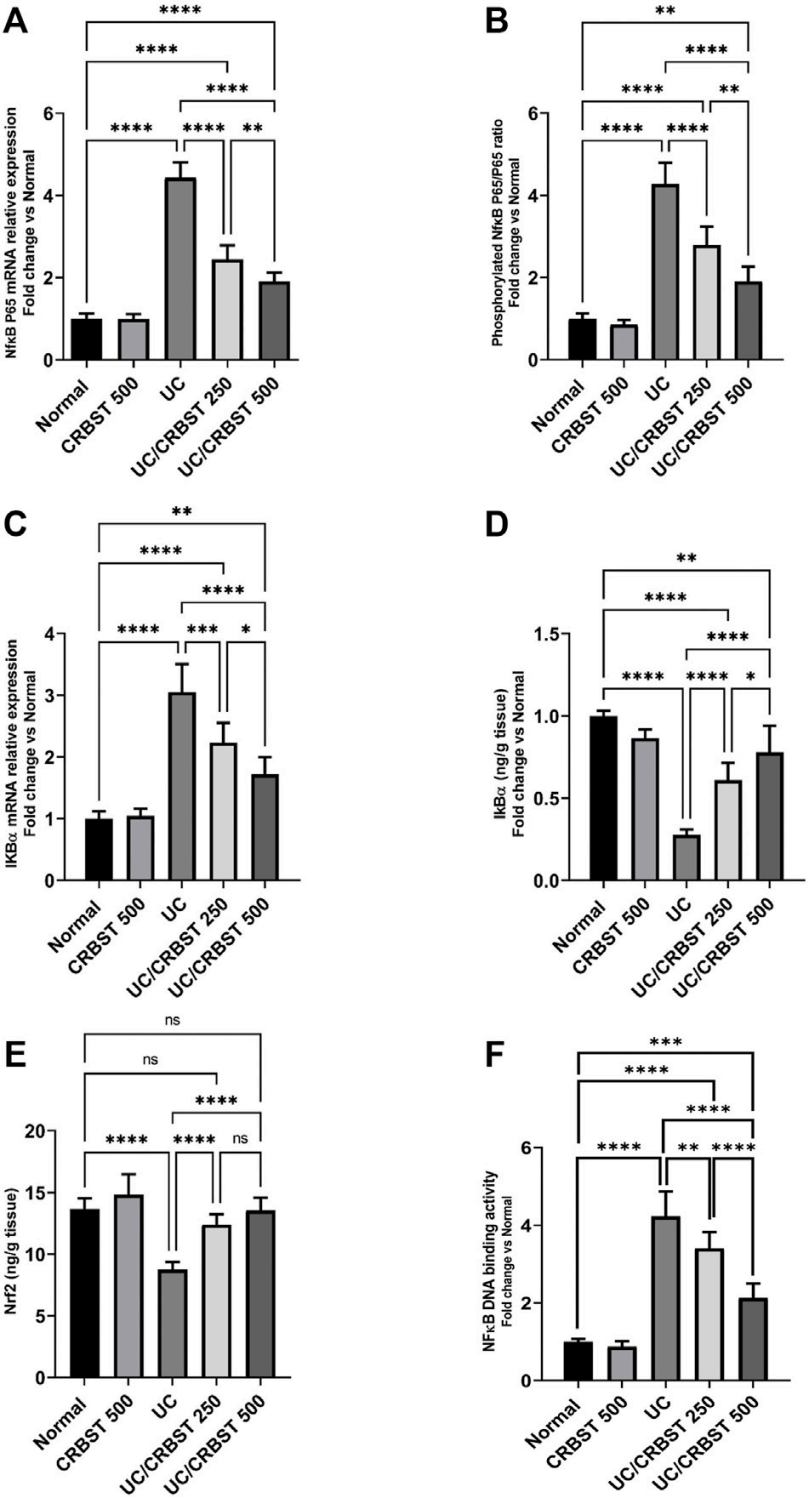
Effect of CRBST 250 and 500 mg/kg on NF-κB p-65 mRNA **(A)**; Phosphorylated NF-κB p-65/p65 ratio **(B)**; IκBα mRNA **(C)**; IκBα **(D)**; Nrf2 **(E)** and NFκB DNA binding activity **(F)** in rats with AA-induced UC. Results are shown as the mean ± SD (n = 6). *p < 0.05, **p < 0.01, ***p < 0.001, ****p < 0.0001. Normal, normal control rats administered the vehicle; CRBST 500, normal rats administered carbocisteine (500 mg/kg); UC, AA-induced UC rats administered the vehicle; UC/CRBST 250, AA-induced UC rats treated with carbocisteine (250 mg/kg); UC/CRBST 500, AA-induced UC rats treated with carbocisteine (500 mg/kg).

Rats with AA-induced colitis showed a marked increase in the NFκB p-65 gene expression (Figure 6A), phosphorylated p-65/p65 ratio (Figure 6B) and IκBα gene expression (Figure 6C); they also showed a significant reduction in IκBα (Figure 6D) and Nrf2 (Figure 6E) protein content when compared with the normal control group. Following the administration of CRBST 250 or 500 mg/kg, a significant reduction in n the NF-κB p-65 gene expression, phosphorylated p-65/p65 ratio and IκBα gene expression was observed, with concomitant increase in the IκBα and Nrf2 protein content. Treatment with CRBST 500 mg/kg was found to be superior to CRBST 250 mg/kg in inhibiting NFκB signaling. These findings were confirmed by the determination of NFκB nuclear DNA binding activity (Figure 6F). In this regard, both doses decreased the NFκB nuclear DNA binding activity as an indication for its nuclear translocation and activation.

## Discussion

Ulcerative colitis is a chronic, idiopathic inflammatory disorder that produces dysfunction in the colon (Torres et al., 2012; Høivik et al., 2013). It is characterized by recurrent and remitting mucosal inflammation that begins in the rectum and progresses to the colon’s proximal segments (Peyrin-Biroulet et al., 2015).

Aminosalicylates are the primary therapeutic option for mild to moderate UC, whereas systemic and topical steroids can be used to treat ulcerative colitis flares. Immunosuppressants and biological medicines are utilized for moderate to severe illness. The relative efficacy of these competing therapy is unknown, and some of them are accompanied with considerable side effects (Navaneethan and Shen, 2010). Eventually, colectomy is required in up to 15% of UC patients (Magro et al., 2012). As a result, further therapeutic strategies based on newly recognized mechanisms should be developed to improve efficacy while avoiding side effects. The current study aims to assess the potential protective impact of CRBST, a safe and extensively used mucoregulatory medication, in UC based on its previously established anti-oxidant and anti-inflammatory properties (Ishibashi et al., 2001; Zheng et al., 2008; Rahman and Macnee, 2012).

AA-induced colitis is one of several experimental colitis models. It is performed in rats by injecting acetic acid into the rectum to cause inflammation and ulceration in the rectum and colon (Randhawa et al., 2014). The damage in this model is associated with epithelial necrosis and edema that permeate the intestinal mucosal layer with respect to the dose and duration of AA exposure (Nakao et al., 2014). The initial injury in this model was a relatively mild epithelium necrosis and edema that varied depending on the amounts and length of AA exposure (Niu et al., 2013).

In agreement with the previous findings, AA-administration in UC group in the current study resulted in a noticeable increase in DAI, MDI, and colon weight/length ratio, suggesting a colonic injury and inflammation. Furthermore, histopathological observations were performed to validate the macroscopic findings; AA was found to produce a marked damage to the structure of colonic tissue and resulted in noticeable pathological alteration including edema, complete necrosis of the crypts and inflammatory cell infiltration. Notably, CRBST treatment has been shown to significantly improve the elevated levels of the macroscopic findings with concomitant improvement in histopathological findings. We revealed restoration of architecture, decreased superficial ulceration of the intestinal mucosa, and decreased inflammatory cell infiltration indicating an overall improvement in disease status.

Previous research has yielded a plethora of scenarios regarding the various mechanisms involved in colitis (Bahrami et al., 2022), with oxidative stress being demonstrated to play an important role (Pereira et al., 2015). During chronic and acute intestinal inflammation, macrophages and neutrophils destroy local tissue by secreting tissue degrading enzymes and reactive oxygen species (ROS) (Rieder et al., 2007). Excess ROS metabolites are also known to be produced by AA-induced colitis. The infiltrated and activated neutrophils are significant sources of these ROS (Millar et al., 1996). ROS react with the majority of biological macromolecules in the cell membrane, including proteins, DNA, and polyunsaturated fatty acids, resulting in lipid peroxidation and subsequent cellular damage and dysfunction. Oxidative stress happens when the balance between pro-oxidative enzymes and the activity of anti-oxidative enzymes is disrupted (Abdelhamid et al., 2018). Cells, fortunately, have evolved many antioxidant defense mechanisms (such metabolites, vitamins, and enzymes) to neutralize or minimize the damaging effects of ROS and/or their byproducts. HO-1 is an antioxidant enzyme that is normally expressed at low levels in most tissues/organs except the spleen; however, it is highly inducible in response to a variety of stimuli to protect cells from oxidative and inflammatory injury (Ryter et al., 2006; Wu et al., 2011). It is up-regulated by NF-κB and other transcription factors in response to oxidative stress and hypoxia (Wu et al., 2004; Hayden and Ghosh, 2008). Additionally**,** exposure to oxidants interrupts the interaction between Keap1 and Nrf2, subsequently, Nrf2 is translocated into the nucleus to induce the transcription of antioxidant proteins, such as HO-1 and catalase (CAT) (Ray et al., 2012; Albert-Garay et al., 2022). Functions of HO-1 include anti-inflammatory, antiapoptotic, antiproliferative, and immunomodulatory effects (Araujo et al., 2012). While, Catalase is another important antioxidant enzyme that helps to reduce oxidative stress by eliminating cellular hydrogen peroxide and producing water and oxygen. (Nandi et al., 2019). Accordingly, in the current investigation, tissue levels of HO-1 were increased in the UC group in response to AA insult. Additionally, a significant decrease in catalase level and TAC content was observed in the UC group’s colon tissue homogenates, indicating that the anti-oxidative enzymes are consumed by AA-induced ROS. CRBST treatment resulted in a significant increase in catalase and TAC in the CRBST-treated groups, indicating a reduction in oxidative stress and this could be attributed to the observed upregulation in HO-1 expression in both the gene and protein levels. In this regard, CRBST treatment resulted in a significant increase in the anti-oxidative enzyme, HO-1. A significant decrease in the highly oxidative enzyme, MPO was also observed. The elevated levels of MPO were previously linked to increased oxidative stress and inflammation (Ndrepepa, 2019; Saber et al., 2021a). MPO also acts as a mediator of oxidative stress by increasing the generation of ROS and reactive nitrogen species (Chen et al., 2020).

According to the current findings, CRBST has exceptional free radical scavenging activities and can counteract the decrease in antioxidant enzyme activity. In accordance with the present results, previous *in vitro*, *in vivo* and clinical research have shown that CRBST can act as an antioxidant by decreasing oxidative stress and boosting antioxidative enzymes, independent of recognized mucolytic activity (Macciò et al., 2009; Nogawa et al., 2009; Choudhury and Macnee, 2017; Catanesi et al., 2021).

Inflammation is another pathogenic scenario implicated in AA-induced colitis (Saber et al., 2021c; Bahrami et al., 2022). There is a close relationship between inflammation and oxidative stress (Abdelhamid et al., 2021a; Abdelhamid et al., 2021b), where oxidative stress is connected to UC exacerbation by stimulating the overexpression of proinflammatory proteins, hence increasing inflammatory responses (Bahrami et al., 2022). The NFκB signaling pathway has been reported to be activated by AA-administration (Viennois et al., 2012; Rezayat et al., 2018; Rashidian et al., 2019). Growth factors, oxidative stress-related enzymes, and cytokines are overexpressed when NFκB is activated (Khalil et al., 2020; Abd El-Fattah et al., 2022a; Abdelhamid et al., 2022; Abd El-Fattah et al., 2022b). Furthermore, this activation influences physiologic or pathologic events including immunological and inflammatory responses. Furthermore, the translocation of NFκB from the cytoplasm to the nucleus, as well as its binding to DNA, results in the transcription of inflammatory mediators such as IL-6 and TNF-α (Liu et al., 2017). The buildup of these inflammatory mediators is important in the development of UC because it alters the balance of proinflammatory and anti-inflammatory molecules (Liu et al., 2017). Thus, inhibiting the inflammatory response is a viable method for treating AA-induced colitis.

In the current investigation, we found that AA administration resulted in a noticeable decrease in IκBα and an increase in NFκB level, which leads to a rise in IL- 6, and TNF-α levels and reduction of IL-10. On the other hand, treatment with CRBST increased IκBα content and decreased the NFκB level which led to suppression of inflammatory cytokines, TNF-α and IL-6, expression and increased IL-10 content. These results are consistent with those of Pena et al. (1999) who reported that CRBST was found to decrease the production of pro-inflammatory cytokines, TNF-α and IL-6. Also, in the study of Wang et al. (2016) on human alveolar epithelial cells, CRBST effectively suppressed inflammation *via* suppressing NFκB signaling pathways. These findings imply that CRBST has an anti-inflammatory effect on AA-induced UC by suppressing the NFκB signaling pathway.

NFκB activation also induces epithelial cell apoptosis and aids in the development of UC (Qiu et al., 2011). Our findings showed that apoptosis was activated in colonic tissues subjected to AA in the UC group, as indicated by upregulation of the pro-apoptotic protein, Bax, and downregulation of the anti-apoptotic protein, BCL-2, resulting in a significant increase in the Bax: BCL-2 ratio when compared to the normal group. Increased apoptosis of epithelial cells most likely leads to epithelial barrier disruption, which contributes to intestinal damage (Becker et al., 2013). It has been found that oxidative stress causes the expression of numerous genes that are important for cellular death via apoptosis (Crespo et al., 2012). Our data revealed that CRBST decreased pro-apoptotic Bax with upregulation of BCL-2, indicating attenuation of colonic apoptosis that was confirmed further by decreased active caspase-3.

Since excessive exposure of intestinal mucosa to ROS under inflammatory stimuli enhances epithelial apoptosis, the suppression of colonic apoptosis can be linked to the observed attenuation of oxidative stress (Kruidenier et al., 2003). Oxidative stress triggers the production of proinflammatory proteins regulated by NFκB, resulting in an inflammatory response. Furthermore, NFκB-mediated inflammation is linked to an increase in ROS production. The link between oxidative stress and inflammatory reactions in UC has received a lot of attention (Tian et al., 2017). Previous preclinical and clinical research have shown that CRBST has powerful antioxidant and anti-inflammatory properties in several organs and disease models (Macciò et al., 2009). These studies, together with our findings, show that CRBST helps to modulate the interaction between oxidative stress and inflammation.

Nrf2 and NF-κB are the two key transcription factors that regulate cellular responses to oxidative stress and inflammation respectively. Pharmacological and genetic studies indicate functional cross-talk between these two critical pathways. The absence of Nrf2 can exacerbate NF-κB activity leading to increased cytokine production, whereas NF-κB can modulate Nrf2 transcription and activity (Wardyn et al., 2015). The Nrf2 transcription factor is involved in the activation of the cellular anti-oxidative and anti-inflammatory defense systems (Lee, 2017; Dias et al., 2020). Many downstream proteins are linked to Nrf2, including HO-1, which plays a role in increasing resistance capacity and suppressing redox flares (Lee, 2017). Thus, we evaluated the effect of CRBST on Nrf2 expression in order to investigate its antioxidant and anti-inflammatory properties, and we discovered that AA administration in the UC group significantly reduced Nrf2 expression. Our findings are consistent with prior research that found that AA exposure reduced Nrf2 expression (Yalniz et al., 2012; Alsharif et al., 2022). When compared to the UC group, there was a significant increase in Nrf2 after CRBST treatment. These findings are consistent with those of Yageta et al. (2014) and Catanesi et al. (2021), who investigated the effect of CRBST and discovered that it has the potential to boost Nrf2 expression.

**FIGURE 7:**
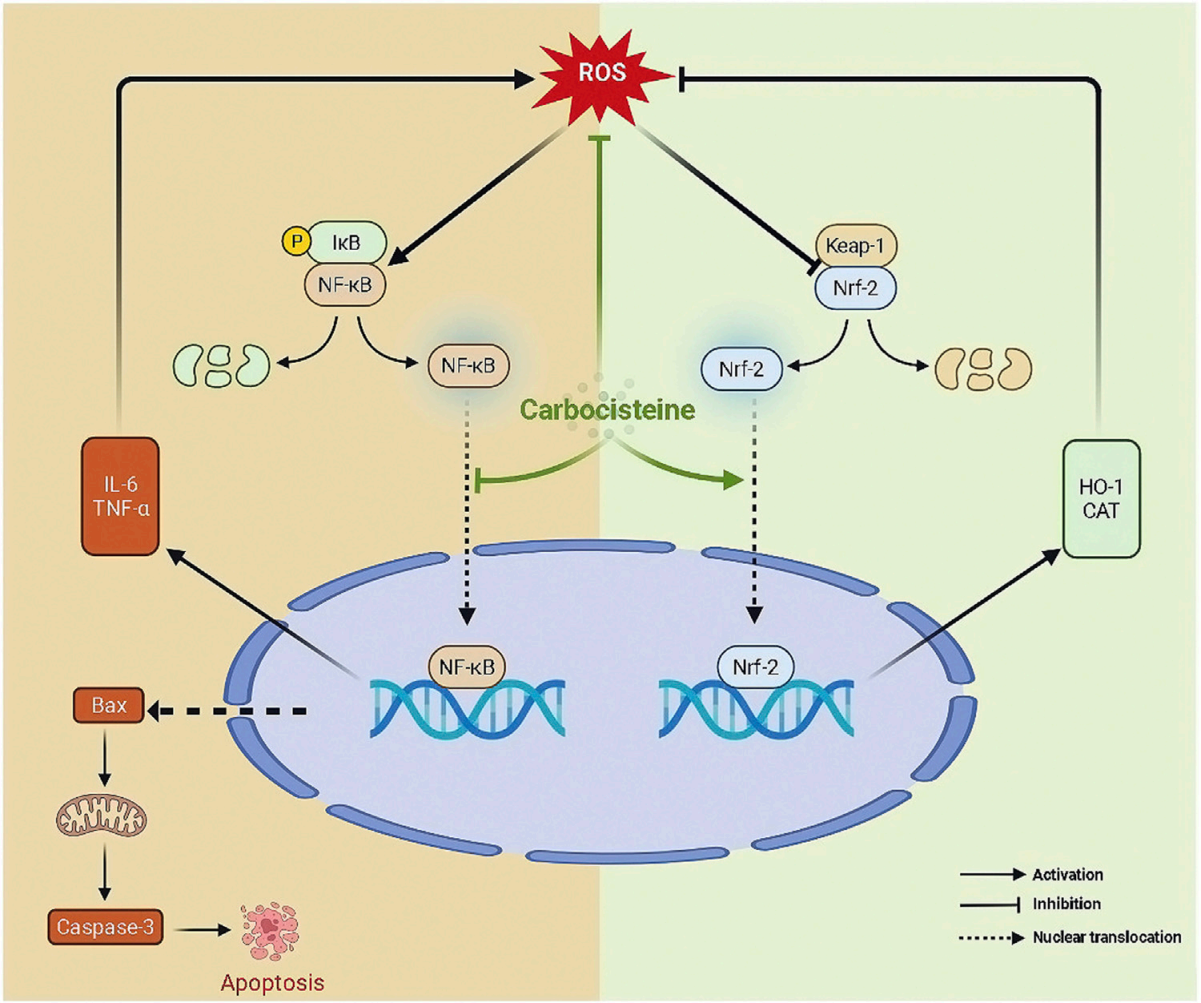
The proposed mechanism of action of carbocisteine.

To conclude, as depicted in Figure 7, carbocisteine, at doses that are quite higher than the human effective dose, inhibited oxidative stress, inflammatory response, and apoptosis in acetic acid-induced colitis in rats through activating Nrf2 and suppressing NFκB. The current study provides a potential basis for repurposing the safe and commonly used mucoregulator, carbocisteine, for the treatment of UC. However, further research into other pathways that run parallel to those proposed in the current study is required to assess the reliability of carbocisteine as a treatment for UC.

## Data Availability

The original contributions presented in the study are included in the article/supplementary material, further inquiries can be directed to the corresponding author.
